# Dynamic Gene Expression Mitigates Mutational Escape in Lysis-Driven Bacteria Cancer Therapy

**DOI:** 10.34133/bdr.0049

**Published:** 2024-09-19

**Authors:** Filippo Liguori, Nicola Pellicciotta, Edoardo Milanetti, Sophia Xi Windemuth, Giancarlo Ruocco, Roberto Di Leonardo, Tal Danino

**Affiliations:** ^1^ Department of Physics, Sapienza University of Rome, Rome, Italy.; ^2^ Center for Life Nano- & Neuro-Science, Istituto Italiano di Tecnologia, Rome, Italy.; ^3^ NANOTEC-CNR, Soft and Living Matter Laboratory, Institute of Nanotechnology, Rome, Italy.; ^4^ Department of Biomedical Engineering, Columbia University, New York, NY, USA.; ^5^ Herbert Irving Comprehensive Cancer Center, Columbia University, New York, NY, USA.; ^6^ Data Science Institute, Columbia University, New York, NY, USA.

## Abstract

Engineered bacteria have the potential to deliver therapeutic payloads directly to tumors, with synthetic biology enabling precise control over therapeutic release in space and time. However, it remains unclear how to optimize therapeutic bacteria for durable colonization and sustained payload release. Here, we characterize nonpathogenic *Escherichia coli* expressing the bacterial toxin Perfringolysin O (PFO) and dynamic strategies that optimize therapeutic efficacy. While PFO is known for its potent cancer cell cytotoxicity, we present experimental evidence that expression of PFO causes lysis of bacteria in both batch culture and microfluidic systems, facilitating its efficient release. However, prolonged expression of PFO leads to the emergence of a mutant population that limits therapeutic-releasing bacteria in a PFO expression level-dependent manner. We present sequencing data revealing the mutant takeover and employ molecular dynamics to confirm that the observed mutations inhibit the lysis efficiency of PFO. To analyze this further, we developed a mathematical model describing the evolution of therapeutic-releasing and mutant bacteria populations revealing trade-offs between therapeutic load delivered and fraction of mutants that arise. We demonstrate that a dynamic strategy employing short and repeated inductions of the *pfo* gene better preserves the original population of therapeutic bacteria by mitigating the effects of mutational escape. Altogether, we demonstrate how dynamic modulation of gene expression can address mutant takeovers giving rise to limitations in engineered bacteria for therapeutic applications.

## Introduction

With rapid advances from synthetic biology, genetically engineered bacteria are emerging as a new alternative for cancer therapy [1,2]. Systemically administered microbes have shown the natural ability of colonizing hypoxic niches in solid tumors, exploiting their immunosuppressive environment [3–5]. Engineering bacteria to produce and release therapeutic molecules in these niches is a promising strategy to deliver payloads into areas of the body that are difficult to reach by traditional approaches. Two important considerations in recent work include (a) designing the optimal therapeutic molecules to be delivered and (b) engineering the bacteria to specifically release therapeutics in space, without affecting healthy tissues and, in time, employing genetic circuits to program pharmacokinetics [6].

For (a), both direct effectors to kill cancer cells [7,8] and immunomodulators [9,10] have been actively explored. A promising category of proteins falling in the former group are bacterial pore-forming toxins (PFTs). Structural studies have revealed that PFTs share the same structure as other protein domains that have been described in the mammalian immune system, involved in mechanisms of bacterial and membrane attack and leading to the rapid killing of pathogen-infected or cancerous cells by the immune system [11]. The growing interest in the characterization of the structure and functions of PFTs has suggested their clinical use as cancer cell therapeutics for several decades [12]. The archetypal example of PFTs is Perfringolysin O (PFO), belonging to the family of cholesterol-dependent cytolysins (CDCs). PFO is produced by the anaerobic bacterium *Clostridium perfringens* and is commonly referred to as Theta toxin [13,14].

**Fig. 1.**
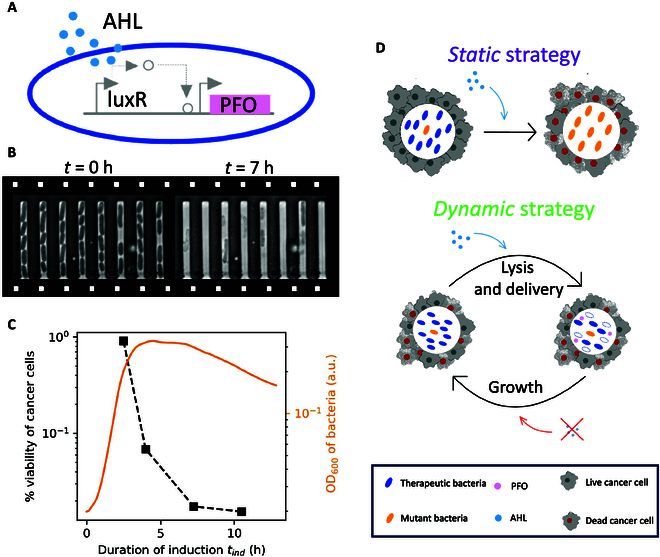
Conceptual framework for the dynamic control of gene expression in lysis-driven bacteria cancer therapy. (A) Schematic representation of the genetic circuit we employed to engineer lysis-driven therapeutic bacteria. (B) Two example images of therapeutic bacteria in a mother-machine before and after 7 h of static induction with 100 nM AHL. (C) OD_600_ of the bacteria in a typical plate reader experiment with 100 nM AHL induction (orange curve), compared to the concurrent viability of cancer cells cultured in bacterial supernatant as a function of the duration of induction (black curve, corresponding error bars are comparable to symbol size, see Materials and Methods for the protocol). The cancer cell line used in the experiment is murine colorectal cancer line CT26. (D) Schematic evolution of the populations of therapeutic and mutant bacteria in the tumor core for a “static” (top) and “dynamic” (bottom) induction of the toxin. See legend on the bottom of the figure.

Recently, in the context of bacteria cancer therapy, bacteria have been shown to achieve cancer killing upon expression of PFO for both co-culture with multicellular spheroids in vitro [15] and intratumoral injection in vivo in non-obese-diabetic scid gamma mice [16]. To the best of our knowledge, previous literature does not report evidence of the mechanism of action of these therapies. In this paper, we used an acyl-homoserine lactone (AHL)-inducible promoter (PluxI) as a remote triggering mechanism for PFO expression in *Escherichia coli* [15] (Fig. 1A) and we demonstrated that the release of PFO occurs through lysis of bacterial vectors from the cytoplasm (Fig. 1B). Lysis is a particularly potent release mechanism in bacteria cancer therapy because it offers 2 additional beneficial effects: (a) it results in the release of bacterial adjuvants capable of triggering immune responses, and (b) it contributes to the controlled reduction of the growth of engineered bacteria. However, a fundamental limitation of bacterial therapies employing a protein-triggered lysis mechanism can be the appearance of nonfunctional mutations in the gene coding for the lysis-triggering protein, due to selective pressure [17].

A key advantage of bacterial cancer therapies with respect to traditional therapeutics resides in the fact that engineered bacteria can colonize the tumor core and proliferate in its immunosuppressive environment. As long as the bacteria grow, the therapeutic molecule can be synthesized by the genetic machinery of the bacterial cells and released on-demand. In fact, it has been shown that multiple delivery cycles in lysis-driven bacteria cancer therapy can be attained, in a system known as the Synchronized Lysis Circuit (SLC) [18]. Here, bacteria form a reservoir of therapeutic in the tumor core, which can be released on-demand upon induction of the lysis gene. However, if therapeutic bacteria are replaced by nonlysing mutants, the reservoir of therapeutic in the tumor core is lost and multiple cycles of delivery cannot be attained. This is an issue reported in the literature also for the case of SLC grown in batch culture [19].

In this paper, we suggest that a dynamic strategy of induction can be employed to preserve the reservoir of the therapeutic, when compared to a static, prolonged induction strategy. This was accomplished by mitigating the impact of mutational escape through multiple cycles of lysis and growth (Fig. 1D). We developed an Ordinary Differential Equation (ODE) model to quantitatively describe the observed population dynamics of *E. coli* expressing the chemically inducible *pfo* gene [20,21]. We leveraged the ODE model to demonstrate that employing dynamic strategies to control gene expression (and therefore therapeutic release) via genetic circuits is an approach to mitigate the detrimental effects of mutational escape and further enhance the advantage of bacteria cancer therapies.

## Materials and Methods

### Bacterial strains, plasmids, and culturing

The host strain used in this study was *E. coli* MG1655. The strain was transformed with plasmid pTD103_theta (origin of replication colE1, antibiotic resistance kanamycin) and a plasmid expressing constitutive green fluorescent protein (GFP) (psc101, chloramphenicol, Fig. S1). All bacteria were grown with appropriate antibiotics for selection (50 μg/ml kanamycin and 25 μg/ml chloramphenicol) in lysogeny broth (LB) media (Sigma-Aldrich) at 200 rpm or on LB-agar plates containing 1.5% agar at 37 °C.

### Plate reader experiments of growth curves

**Fig. 2.**
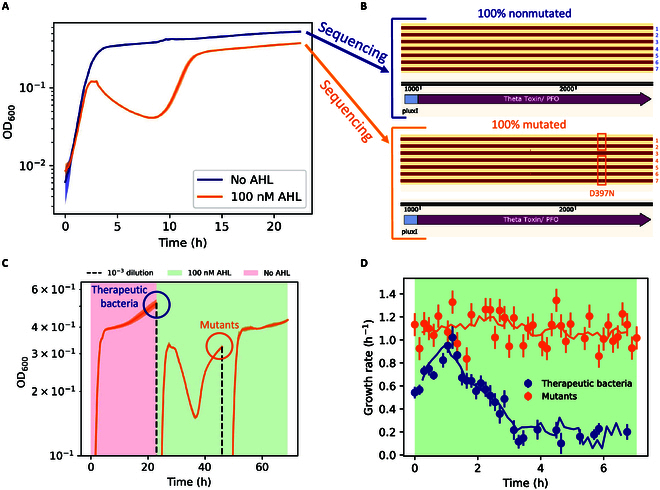
Mutational escape for the therapeutic bacteria. (A) Growth curves measured in the plate reader of therapeutic bacteria grown without AHL (blue curve) or with 100 nM AHL (orange curve). The shaded area represents the standard deviation of a technical triplicate. (B) Sequences of plasmids extracted from bacteria grown with and without AHL, aligned to pTD103_theta (Fig. S1). The alignment is performed with the Snapgene software. For mutants, the slits highlighted with orange squares in aligned sequences are point mutations, while the thin vertical bar above aligned sequence 3 indicates a point insertion. (C) Growth curves of the bacteria growing in medium with varying concentrations of AHL, diluted twice from stationary phase. The plot shows that mutant bacteria lose the ability to lyse. (D) Growth rate of therapeutic and mutant bacteria in the presence of 100 nM AHL, as measured experimentally in a microfluidic mother-machine. The curve for the therapeutic bacteria is initially increasing because bacteria are seeded in the mother-machine from the overnight culture. The solid lines represent a running average of *n* = 5 data points. The error bars represent the standard deviation of the growth rates computed for all individual cells in the mother-machine at each time point.

Each experiment started with dilution to OD_600_ (optical density at 600 nm) 0.01 of overnight culture in fresh LB with appropriate antibiotics (refresh). A 96-well plate was loaded with 200 μl of refreshed bacteria in each well and the appropriate concentration of AHL. The samples were incubated at 37 °C with shaking in a TECAN Infinite M Nano+ plate reader, and the OD_600_ was measured every 10 min. For the experiment shown in Fig. 4B, the Lab Automation Robot Opentrons OT-2 was programmed with a custom-written Python code to transfer appropriate volumes of AHL to the wells of the plate in order to achieve triplicates with logarithmically spaced concentrations. Dilutions in plate reader experiments (Fig. 2C) were performed by stopping the kinetic cycle in the plate reader, diluting the bacteria from each well in another well of the plate, with fresh LB, appropriate antibiotics and concentrations of AHL, and starting a new kinetic cycle.

### Detection of mutants through sequencing

After growth to stationary state over 23 h with appropriate concentrations of AHL, bacteria were diluted 1:10^6^ and plated on an agar plate with antibiotics and no AHL. Clear distinct colonies were observed on the plates. Seven colonies from each plate were grown overnight and the plasmids extracted from those clones were sequenced via Plasmidsaurus. Since pTD103_theta has a higher copy number than the constitutive GFP plasmid, the main peak from sequencing reads corresponded to the plasmid containing *pfo*. We aligned the sequencing reads through Snapgene with the original sequence of pTD103_theta to detect mutations in the *pfo* gene.

### Prediction of the fraction of mutants through fitting with simulations of the ODE model in Eq. 1

The fraction of mutants in a culture at a fixed time point was predicted through fitting with the simulations of the ODE model in Eq. 1. The culture of interest was diluted to OD 0.01 in LB with appropriate antibiotics and 100 nM AHL. Three replicates of 200 μl of the diluted bacteria were loaded in the well plate and the growth curves were measured. The growth curves were fitted to the simulations, with the only unknown parameters being *x*_0_ and *y*_0_, from which the fraction of mutants of the initial culture was retrieved.

### Measurement of the release of GFP in the supernatant

The cultures were diluted to OD 0.01 from overnight in 3 replicates of 10 ml of LB (supplied with antibiotics and 100 nM AHL) and incubated at 37 °C and 200 rpm. For each time point, a sample of 500 μl of the growing culture was collected and transferred to a 1.5-ml microcentrifuge tube. The sample was centrifuged in a table-top centrifuge at 3,000 rpm for 5 min, and the supernatant was collected and passed through a 0.22-μm filter. GFP was measured in the TECAN plate reader (excitation 488 nm, emission 510 nm), loading the wells with 200 μl of the filtered sample.

### MTT assay of viability

The supernatant was collected as described above and stored at 4 °C until the last time point (∼10 h from induction). CT26 murine cancer cells were cultured with Roswell Park Memorial Institute (RPMI) medium in a T75 flask at 37 °C and 5% CO_2_. When they reached 80% confluence, they were passaged and loaded into the well plate (25,000 cells per well). Cells were grown overnight in 100 μl of RPMI to allow adhesion to the plate. The next day, the medium in the wells (100 μl total volume of the culture medium in each well) was changed to RPMI supplied with 20% supernatant (3 replicates for each time point of collection) and 1.5 μg/ml gentamicin, and the plate was incubated at 37 °C and 5% CO_2_ for 24 h. Viability of the cells was measured with a Sigma-Aldrich In Vitro Toxicology Assay Kit, MTT based (TOX1). The 565-nm absorbance data were normalized with the negative control (RPMI supplied with 20% LB and gentamicin).

### Mother-machine fabrication

Master molds with mother-machine features were fabricated using a hybrid technique involving standard soft lithography and 2-photon polymerization, as described in Cannarsa et al. [22]. Briefly, the feed channel (50 μm wide, 15 μm high) was fabricated on an SU8 layer using standard 2-dimensional lithography protocols. Then, a second layer of 20 μm was spun over the feed channel and, on this second layer, micrometer-sized channels were fabricated on the edge of the larger channel by direct laser writing using a custom 2-photon polymerization setup [23]. Finally, the master mold was silanized to prevent the PDMS from adhering to the master. The PDMS chips were prepared from a Sylgard 184 silicone elastomer kit (polymer base and curing agent were mixed in a ratio of 10:1) and bonded to the glass by oxygen plasma treatment.

### Mother-machine experiment and analysis

From a frozen glycerol stock at −80 °C, bacteria were cultured overnight in LB and appropriate antibiotics for 16 h in a shaking incubator at 37 °C and 200 rpm. From this culture, 1 ml of culture was centrifuged in an Eppendorf mini Spin Plus centrifuge at 1,000 rcf for 5 min and concentrated 50×. The concentrated bacteria were loaded with a pipette into the feed channel of the chip. Then, the entire chip was centrifuged for 7 min at 1,300 rpm to fill the micrometer-size channels of mother-machine (Eppendorf 5430R centrifuge with Combislide adapter). For the duration of the experiments, the chip was perfused with LB with antibiotics and 0.1% bovine serum albumin at a constant flow of 5.5 μl/min using a syringe pump, with a higher initial flow at the beginning of the experiment to remove excess bacteria (50 μl/min for 5 min).

Phase contrast and fluorescence images were acquired every 3 min using a custom-built optical microscope equipped with a 100× magnification objective (Nikon MRH11902; NA = 1.3) and a high-sensitivity complementary metal–oxide–semiconductor (CMOS) camera (Hamamatsu Orca-Flash 4.0 V3). More details on the microscope setup can be found in Cannarsa et al. [22]. Individual cells were then segmented from the fluorescence images using the pretrained neural network model Cellpose 2.0. [24], which we further trained with our data to increase the accuracy of the segmentation. The masks obtained after segmentation were used to measure the number of bacteria and the average growth rate of all cells as a function of time. For the measurement of the growth rate, we used a customized python code that can track the lineage of bacteria in the mother-machine by identifying cell division events; mother, daughter, and neighboring cells based on the area of the bacteria; and the position of their center of mass.

### Molecular dynamics simulations

The simulations were performed using Gromacs 2021.6 [25] using the CHARMM-36 force field [26]. Proteins were placed in a dodecahedric simulative box, with periodic boundary conditions. The TIP3P model for water molecules [27] was used. In both systems, all protein atoms were at least at a distance of 1.1 nm from the box borders. The minimizations were performed with the steepest descent algorithm. Next, a 2-step thermalization of the system was run in NVT and NPT environments each for 0.1 ns at 2-fs time steps. The temperature was kept constant at 300 K by using the v-rescale thermostat, while the pressure was set at 1 bar with the Parrinello-Rahman barostat [28]. We adopted the LINCS algorithm [29] to constrain bonds involving hydrogen atoms. Short-range nonbonded interactions were evaluated with a cutoff of 12 Å. The Particle Mesh Ewald method [30] was adopted for the long-range electrostatic interactions.

### Simulations

All simulations were performed by direct Euler integration of Eq. 1 using the custom Python program. The selection of model parameters was motivated as follows: the value of the parameters *m* = 1, *k* = 19 a.u., and *β*_0_ = 6.1 h ^−1^ was estimated from experiments (Fig. S9 and Fig. 4B). The value of the degradation of intracellular PFO is set a priori to *δ* = 0.1 h ^−1^ (in the range of slow-decaying proteins [31]), because the length of the experiment does not reveal such long degradation times. All other parameters are set as the mean value of the fitted parameters of the model in Eq. 1 with the experimental growth curves in Fig. S7: *γ*_0_ = 1.57 h ^−1^, *ρ_s_* = 0.33 a.u., *α*_0_ = 1.39 h ^−1^, *K* = 3.29 a.u., *n* = 4.46. For the model accounting for substrate consumption (Fig. S10), we used a rate of substrate utilization *r_s_* = 4.17 h ^−1^ to maximize accordance with experimental data in Fig. S10A. For the model accounting for adaptive mutation (Fig. S11), we used a mutation rate *μ* = 10^−7^ h ^−1^, consistently with the literature [32].

## Results

### *E. coli* expressing PFO as a cancer therapy

The genetic circuit we employed (in nonpathogenic *E. coli* MG1655) is an AHL-inducible system based on elements of the quorum sensing machinery in *Vibrio fischeri* [15]. The circuit contains a constitutively expressed LuxR [33], which can bind to autoinducer AHL (Fig. 1A). The complex LuxR-AHL serves as an activator binding to promoter PluxI and inducing expression of PFO. Since the *luxI* gene is not present in the circuit, *E. coli* cannot synthesize AHL on its own and the system relies solely on externally supplied AHL for induction, enabling better control over the protein production rate, in the interest of mathematical modeling. Additionally, the bacteria were transformed with constitutive GFP enabling better imaging for microscopy. We first characterized the mechanism of action of *E. coli* expressing PFO as bacterial cancer therapy. We grew the therapeutic bacteria in a microfluidic device in which cells are trapped in short channels and excess cells are washed away by fresh medium (mother-machine [34]) and we monitored the number of cells as a function of the duration of induction (Materials and Methods). Expression of PFO was induced by adding 100 nM AHL to the growth medium flowing in the chip. We observed clear lysis of the bacterial cells (Fig. 1B and Movie S1) and a decrease in the number of bacteria upon induction of the toxin. We clearly appreciate that after a delay of approximately 4 h, the number of bacteria peaked and then decreased by more than 60% after 10 h (Fig. S2). This suggests that 4 h is the time required for the expression of a sufficient concentration of PFO to trigger cell lysis, in the presence of 100 nM AHL. Then, we moved to characterization of the efficacy of the toxin released upon lysis as a direct promoter of cancer cell death. To achieve this, we first grew the therapeutic bacteria in our plate reader setup (Materials and Methods) with 100 nM AHL, and observed a peak and drop in the OD_600_ of the culture (Fig. 1C, orange curve in the plot), with approximately the same time delay of 4 h in the triggering of lysis that we observed in the microfluidic experiments. We collected the supernatant of the bacterial culture and cultured the murine colorectal cancer cell line CT26 with complete RPMI and 20% supernatant. After incubation for 24 h, we measured the viability of the cells with an MTT viability assay (see Materials and Methods). We observed progressively lower viability of the cancer cells (Fig. 1C, black curve in the plot) with increasing duration of the induction of toxin expression (*t_ind_*). No decrease in viability was observed culturing CT26 cells with the supernatant collected from therapeutic bacteria not induced with AHL (Fig. S3).

These findings demonstrate that *E. coli* expressing PFO can serve as a lysis-driven bacteria cancer therapy, with reproducible time dynamics of lysis and cancer cell killing upon release.

### Mutational escape and population bottleneck for the therapeutic bacteria

Since the induction of PFO triggers bacterial lysis, we reasoned that in a therapeutic setting, this could lead to a population bottleneck for therapeutic bacteria (i.e., the bacteria capable of producing and releasing the therapeutic). Eventually, prolonged induction of the lysis gene can cause the substitution of the original population of bacteria with nonlysing mutant escapees, as outlined in Fig. 1D. To investigate this, we measured growth curves of the bacteria in our plate reader setup for longer inductions of toxin expression, up to 24 h. After initial exponential population growth, we observed a ≈70% drop in OD_600_ starting at time point *t* ≈ 3 h. Later, at time point *t* ≈ 8 h, we observed a resumption of growth as indicated by a rapid increase in OD_600_. Notably, 100 nM AHL is still present in the medium (Fig. S4), indicating that this second wave of growth without apparent lysis is occurring under conditions that would induce the toxin (Fig. 2A, orange curve). To demonstrate that this behavior is owing to mutations, we grew bacteria for 23 h with or without AHL through one round of passaging (see Materials and Methods) and demonstrated that 7 of the 7 resultant AHL-supplemented colonies contained mutations in the plasmid-borne *pfo* gene, while plasmid loss was not observed in any colony. One colony (sequence 3 in Fig. 2B, bottom reads) carried a frameshift mutation in the toxin gene, whereas the other 6 colonies carried the same missense variant (D397N). No mutations were detected in bacteria grown without AHL (Fig. 2B, top reads). We employed a methodology using both experimental assays and computational predictions from molecular dynamics to show that the missense mutation we observed caused loss of function of PFO in lysing the inner membrane of *E. coli*.

To investigate the functional difference between original therapeutic bacteria and mutants, we performed a plate reader experiment with the concentration of AHL in the medium varying in time (Fig. 2C). When the therapeutic bacteria were grown without AHL up to stationary phase and passaged in fresh medium with 100 nM AHL, the OD_600_ curve showed clear lysis in the second part of the experiment, followed by the growth of mutants up to the stationary phase. When the bacteria were once again passaged in fresh medium with 100 nM AHL, the OD_600_ curve of the passaged culture did not show sign of lysis. Functionally, this demonstrates that the mutant population lost the ability to lyse upon toxin induction. We also observed a modulation of the growth rate caused by the wild-type (wt) toxin. This effect was measured experimentally in the mother-machine (see Materials and Methods), where we observed a progressive decrease in the growth rate preceding lysis (Fig. 2D, blue curve, and Movie S2). This modulation was not observed for the case of nonlysing mutants (Fig. 2D, orange curve, and Movie S3).

**Fig. 3.**
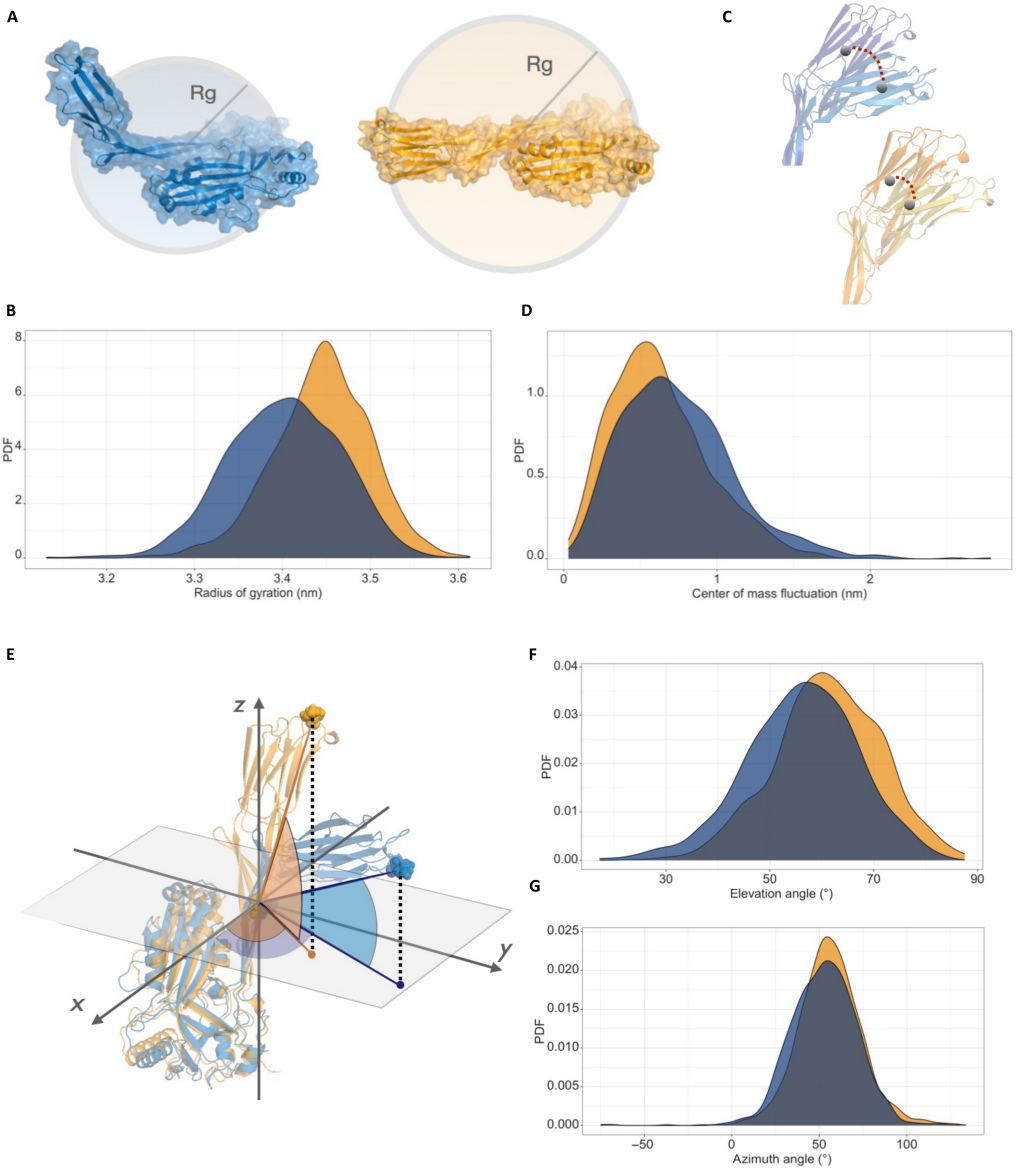
Results of dynamic–structural molecular dynamics simulations of wt Perfringolysin O and mutant form D397N. (A and B) Radius of gyration over the simulation time for wt (blue) and mutant D397N (orange): schematic representation over 2 example simulated protein configurations (A) and distributions (B). (C and D) Fluctuation of the center of mass of the membrane-bound region spanning from residue 391 to 500 [13]: schematic representation over 2 example simulated protein configurations (C) and distributions (D). (E to G) Relative bending of the membrane-bound region with respect to the other region of the protein: schematic representation of the calculated angles (E) and distributions of the elevation angle (F) and azimuthal angle (G). In all figures, blue represents the wt and orange represents the mutant form.

We employed a dynamic–structural computational approach to investigate whether the specific missense mutation we observed inactivates the mechanism of pore formation induced by PFO [35]. To our knowledge, there is no previous literature elucidating the precise mechanism of *E. coli* lysis upon expression of PFO, since the inner membrane of *E. coli* does not contain cholesterol. We considered the hypothesis that the mechanism of action to induce lysis from the inside of *E. coli* is the same as that reported in the literature for canonical PFO pore formation from the outside [36–38]. The mechanism of pore formation involves initial oligomerization of the monomers on the target membrane, followed by a concerted conformational change leading to membrane insertion through *β*-hairpins. This conformational change is caused by rotation and bending toward the membrane of the regions of the protein, which are not bound to the membrane itself. The results of the molecular dynamics simulations are presented in Fig. 3. The wt form was studied considering the structure experimentally resolved using x-ray crystallography (Protein Data Bank code: 1PFO [14]) at a resolution of 2.2 Å, while the mutated form was obtained through computational mutation. In Fig. 3B, we illustrate the distributions of the radius of gyration values over the simulation time (1 μs) for both the wt and D397N, represented in blue and orange, respectively (see Fig. S5 for the time dynamics in the whole 1 μs of the simulations). The plot suggests that the wt can have a more compact structure with respect to the mutant D397N (as schematically depicted in Fig. 3A). Specifically, the radii of gyration for the conformations explored by the dynamics of the wt and mutated form are 3.40 ± 0.06 nm and 3.44 ± 0.05 nm, respectively. To characterize the motion of the wt form, we focused on the motion of the center of mass within the region spanning from residue 391 to residue 500, i.e., the C-terminal region of the protein that binds to the target membrane [39]. On the same note, in Fig. 3D, we present the distributions of the distance between the center of mass of the region spanning from residue 391 to residue 500 at each time point and the mean position of the center of mass. The analysis just reported, as schematically illustrated in Fig. 3C, revealed a greater mobility of the wt in comparison to the mutated form.

As a final analysis, to identify a potential preferred direction of motion, we aligned the structures to a moving frame of reference through principal component analysis and calculated the orientation in space of the membrane-bound region with respect to the other region of the protein. For this purpose, we defined an elevation angle and azimuthal angle to describe the relative orientation of the 2 protein regions. A schematic representation of the calculated angles is shown in Fig. 3E. As shown in Fig. 3F, the wt form of the protein explores conformations characterized by smaller elevation angles from the *xy* plane compared to D397N, indicating a rotational motion of the region 391 to 500 relative to the region 30 to 390, with the pivot around residue 81. Specifically, for the wt form, the average elevation angle is 56° ± 19°, while for the mutated form, it is 61° ± 10°. On the other hand, the azimuthal angle distribution helps identify the direction where protein compactness occurs, bringing regions 30 to 390 and 391 to 500 closer. The average azimuthal angle for both systems is 54° ± 19°.

The molecular dynamics analysis cannot be directly used to draw conclusions on the functional properties of mutant D397N. However, it is an interesting mechanistic complement to the phenomenology of the growth curves shown in Fig. 2C and suggests that the loss of function of mutant D397N in lysing the inner membrane of *E. coli* can be attributed to the reduced mobility and bending of PFO. This reduced mobility would prevent the aforementioned drastic collapse by rotation of the monomers bound to the membrane, disabling *β*-hairpin insertion, subsequent pore formation, and lysis of the bacterial cell. The specific mutation we observed (D397N) is not necessarily the only one inhibiting the lysis efficiency of PFO, but this analysis was aimed at demonstrating that static induction for ∼24 h selects a population of nonlysing mutants from the original population of therapeutic bacteria.

### Modeling the population bottleneck

Following the experimental observations, we built a mathematical model of the population bottleneck. We employed the following system of differential equations to model the evolution of the therapeutic and mutant populations in our system of PFO-expressing bacteria:

$$\begin{array}{ll} \overset{\cdot }{x} & =\left(\gamma -\alpha \right)x\\ \overset{\cdot }{y} & =\gamma y\\ \overset{\cdot }{p} & =\beta \left(\left[\mathrm{AHL}\right]\right)-{\left(\gamma -\alpha \right)}^{+}p-\delta p\\ \gamma & =\gamma_{0}\left(1-\frac{x+y}{\rho_{s}}\right)\\ \alpha & =\alpha_{0}\frac{p^{n}}{K^{n}+p^{n}}\\ \beta & =\frac{\beta_{0}{\left[\mathrm{AHL}\right]}^{m}}{k^{m}+{\left[\mathrm{AHL}\right]}^{m}} \end{array}$$ (1)

where *x* and *y* are the concentrations of therapeutic and mutant bacteria, respectively, and *p* is the concentration of the wt toxin in each identical therapeutic bacterium. The toxin-induced lysis rate *α* is modeled as a Hill function of the concentration of the toxin, with maximum rate *α*_0_, cooperativity *n*, and threshold concentration *K*. The Hill function models the aforementioned observation that oligomerization of PFO is needed for pore formation [12]. The parameter *δ* is the intracellular degradation rate of the toxin, which is negligible for nonzero growth rates *γ* but relevant when bacteria are in stationary phase [40]. The formalism ()^+^ represents the positive part of the function in parenthesis, which prevents negative dilution rates (Fig. S6). The parameter *β* models the [AHL]-dependent toxin production rate in the single bacterial cell. The growth rate of the bacteria, which also determines the dilution rate of the toxin, accounts for the growth rate at exponential phase *γ*_0_ and the carrying capacity *ρ_s_*. The carrying capacity is owing only to cell crowding effects [41,42], while accounting for nutrient depletion is not needed to recapitulate the experimental data (Fig. S10). This is a representative model for tumor-colonizing bacteria in vivo, where blood flows prevent nutrient depletion and the carrying capacity is mainly determined by crowding [43]. See Fig. S10 for comparison with a model explicitly considering substrate consumption in the form of a Monod uptake [44]. Additionally, the model in Eq. 1 does not account for the dependence of the mutant population *y* on the therapeutic strain *x* governed by the adaptive mutation rate *μ*. This was not required to recapitulate the experiments for short time frames, when *μ* is of the order reported in the literature for *E. coli* [32]. See Fig. S11 for comparison with a model explicitly accounting for adaptive mutation. On the other hand, the model in Eq. 1 accounts for the modulation of the growth rate caused by the production of the wt toxin (Fig. 2D), which we found necessary to recapitulate the experimental data.

**Fig. 4.**
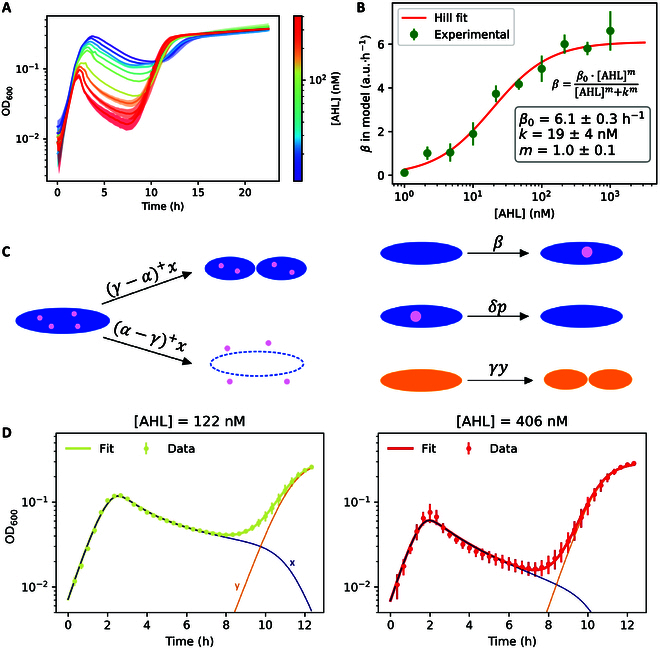
Mathematical model of the population bottleneck fitted to experimental data. (A) Experimental growth curves of the bacteria in plate reader, for different concentrations of AHL in the medium, indicated by the color bar on the right. (B) Values of the parameter *β* in Eq. 1, obtained experimentally with an in vitro characterization of promoter PluxI (Fig. S9), fitted to a Hill activatory function of [AHL]. (C) Schematic interpretation of the model parameters, with the rate of each reaction over the arrow. Blue represents therapeutic bacteria and orange represents mutants. The pink circle represents PFO. (D) Two example experimental curves fitted to computational simulations with the model in Eq. 1. The orange and navy blue thin lines in the plots represent the simulated concentration of mutants (*y*) and therapeutic bacteria (*x*), respectively. All other fits are shown in Fig. S7.

To validate our model, we fitted the parameters of the simulation to the growth curves of the bacteria acquired in plate reader experiments for various concentrations of AHL in the medium (Fig. 4A). For the duration of the plate reader experiment, the concentration of AHL was considered constant and equal to that supplied externally. In fact, bacteria express neither LuxI, which catalyzes production of AHL, nor AiiA, which catalyzes the degradation of AHL [33]. The dependence of the toxin production rate *β* with externally supplied AHL was obtained experimentally with an in vitro characterization of a genetic circuit containing the fluorescent reporter GFP regulated by promoter PluxI, along with *luxR* gene under promoter PluxR (Fig. S9). Given the modular nature of the genetic parts that we employed to build the genetic circuits, the measured GFP time profile is a reliable proxy for the PFO time profile in the system. Indeed, the experimental values of *β* we obtained were fitted by an activatory Hill function of [AHL] (Fig. 4B), with cooperativity (*m* = 1.0 ± 0.1) and induction threshold (*k* = 19 ± 4 nM) consistent with previous characterizations in the literature [45,46]. Two example fits of the growth curves with the model are shown in Fig. 4D, along with the experimental data and the time evolution of therapeutic bacteria *x*(*t*) and mutants *y*(*t*) simulated with the fitted parameters. The values of the fitted parameters and the values of *x* and *y* at time 0 were very consistent when fitting the growth curves for different concentrations of AHL (Fig. S8). The fraction of mutants at *t* = 0 (i.e., the fraction of mutants in our stock) as fitted from all curves in Fig. 4A is equal to (7 ± 6) × 10^−5^.

The system of ODEs we presented fits experimental data for all tested concentrations of the inducer AHL (Fig. S7). It can serve as a platform to model the evolution of therapeutic and mutant bacteria for similar systems, encoding a lysis mechanism that can be triggered upon induction of a gene.

### Dynamic control of toxin production: Experiments and computational predictions

As mentioned, one of the advantages of bacterial therapies resides in the fact that in such systems, the therapeutic molecule is produced by bacteria themselves, by means of the biological machinery orchestrating cell division and gene expression. In the case of the therapy characterized in this paper, when *E. coli* colonizes the core of the tumor, it forms a virtually unlimited reservoir of PFO: as long as bacteria are growing and expressing their genes, the toxin can be produced and released upon induction [47]. The model and data presented in the previous sections highlighted a limitation of this therapy. We showed that prolonged induction of the toxin causes the therapeutic bacteria to be replaced by nonlysing mutants. By appropriately changing the parameters of the model, these findings can be generalized to similar bacterial therapies, employing a mechanism of genetic self-toxicity or lysis of the bacterial vector to deliver the payload. Therefore, upon static induction, the reservoir of therapeutic molecules is lost, and so is one of the key advantages of the bacterial therapy. This is in line with what is shown in Fig. 2C: we can release only one bolus of therapeutic upon static induction (we were not able to release multiple boluses).

**Fig. 5.**
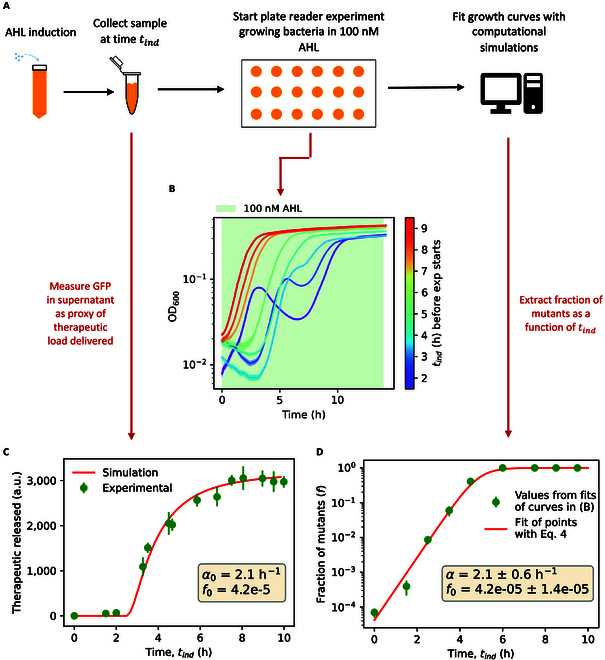
Experimental trade-off between therapeutic load delivered and fraction of mutants. (A) Protocol to measure the amount of mutants and therapeutic bacteria as a function of induction time with AHL (*t_ind_*). (B) Experimental growth curves in the plate reader of bacteria refreshed to OD_600_ 0.01 after being exposed to 100 nM AHL for different induction times (color bar on the right). After refresh, all samples are grown in equal conditions (AHL 100 nM). Shaded area is the standard deviation of a technical triplicate. (C) Therapeutic load released as a function of induction time with AHL. Experimental time points are GFP fluorescence of the supernatant (constitutively expressed and released upon lysis as a genetically encoded cargo). The values of the parameters in the yellow box are used in the simulation (red line, therapeutic released calculated as in Eq. 5). (D) Fraction of mutants as a function of induction time with AHL, extracted by fitting the parameters *x*_0_ and *y*_0_ from the experimental curves in (B). Experimental time points are fitted to the function in Eq. 4. Fitted parameters are in the yellow box.

Controlling the fraction of mutants while releasing a sufficient dose of toxin is therefore paramount to maximize the potency of bacterial therapies. To experimentally quantify these 2 effects for the case of our therapy, we employed indirect approaches to measure the therapeutic load released and the fraction of mutants. The mathematical model in Eq. 1 can be exploited to predict the fraction of mutants in time, *f*(*t*). We developed an experimental protocol to fit from plate reader data the number of therapeutic bacteria (*x*_0_) and mutants (*y*_0_) of a culture of interest (Materials and Methods), as a function of the induction time of toxin expression *t_ind_* (Fig. 5A). Bacteria refreshed to OD_600_ 0.01 in 100 nM AHL, after being previously exposed to 100 nM AHL for different induction times, have markedly different growth curves (Fig. 5B). From these curves, we can extract the parameters *x*_0_ and *y*_0_ by fitting with the model in Eq. 1. The resulting curve of the fraction of mutants as a function of induction time *f*(*t_ind_*) (Fig. 5D, green points) was compared to the time evolution of the fraction of mutants as predicted by the model in Eq. 1:

$$\frac{d}{\mathrm{dt}}\;f\left(t\right)\equiv \frac{d}{\mathrm{dt}}\left(\frac{y}{x+y}\right)=\frac{\alpha \left(t\right)\mathrm{xy}}{{\left(x+y\right)}^{2}}=\alpha \left(t\right)f\left(1-f\right)$$ (2)

Solving the first-order differential Eq. 2 for the fraction of mutants *f*(*t*), we obtain:

$$f\left(t\right)=\frac{1}{\text{exp}\left(C-\int_{0}^{t}‍\alpha \left(t^{\prime }\right){\mathrm{dt}}^{\prime }\right)+1}$$ (3)

where the constant $C=\text{ln}\left[\;f_{0}^{-1}-1\right]$ and *f*_0_ ≡ *f*(*t* = 0).

In order to fit the experimental data with this function (Fig. 5D), we considered the approximation of a constant lysis rate *α*(*t*) ≡ *α*:

$$f\left(t\right)=\frac{e^{\alpha t}}{f_{0}^{-1}-1+e^{\alpha t}}$$ (4)

The resulting values of the fitted parameters [lysis rate *α* = 2.1 ± 0.6 h ^−1^ and fraction of mutants in the stock *f*_0_ = (4.2 ± 1.4) × 10^−5^] are consistent with the previous fits of the experimental growth curves presented in Fig. 4 and Fig. S8. Notably, with these parameters, the fraction of mutants after 5 h of static induction is higher than 10^4^ times the initial fraction of mutants *f*_0_.

We hypothesized that an effective strategy to mitigate the population bottleneck for therapeutic bacteria involves implementing repeated and short inductions (dynamic strategy) rather than a prolonged induction (static). The obvious drawback to this strategy is that as the induction duration shortens, there is a concurrent decrease in the amount of toxin released, reflecting a lower number of therapeutic bacteria lysed. In our system, GFP is constitutively expressed as a genetically encoded cargo; therefore, we used the fluorescence of GFP in the supernatant as a proxy for the amount of toxin released in the medium upon lysis. The proportionality factor depends on the copy number of the corresponding plasmids and the strength of the corresponding promoters, as well as the fluorescence quantum yield. The experimental curve of the therapeutic load released as a function of the length of induction *t_ind_* (Fig. 5C, green points) confirms the trade-off between the therapeutic load delivered and the fraction of mutants. From the simulations, we can use the total number of therapeutic bacteria lysed (Fig. 4C) as a proxy for the therapeutic load delivered (Eq. 5):

$$\text{total toxin released}\propto \int_{t^{\prime }=0}^{t^{\prime }=t}‍{\left(\alpha \left(t^{\prime }\right)-\gamma \left(t^{\prime }\right)\right)}^{+}x\left(t^{\prime }\right){\mathrm{dt}}^{\prime }.$$ (5)

The experimental values of the therapeutic load delivered are consistent with the predictions of the model in Eq. 1 (Fig. 5C, red line), simulated with parameters *α*_0_ and *f*_0_ equal to those fitted as in Fig. 5D.

**Fig. 6.**
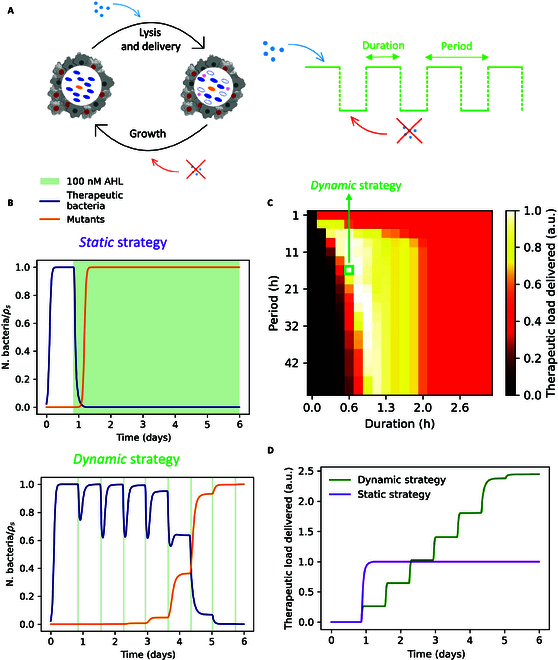
Model prediction of a dynamic strategy of induction to mitigate mutational escape. (A) Schematic of the dynamic strategy of induction with AHL (see the legend from Fig. 1D). (B) Simulated time evolution of therapeutic bacteria and mutants for a static induction (top) and an example dynamic induction (bottom), normalized for the carrying capacity *ρ_s_*. The parameters are the same as those in the simulation shown in Fig. 5C. (C) Heat map showing the amount of toxin released before mutant takeover for dynamic strategies with varying periods (*y* axis) and duration of induction (*x* axis). (D) Simulated therapeutic load delivered as a function of time for the 2 strategies in (B), normalized to the maximum value for the static strategy.

Ultimately, we leveraged the model in Eq. 1 to get a sense of how short and frequent these inductions have to be to maximize our desired effect. As time programs of induction with AHL, we simulated square waves with varying periods and duty cycles inducing repeated cycles of lysis (delivery) and growth (dilution of intracellular toxin in therapeutic bacteria) (Fig. 6A and B).

The simulations predict that tuning the parameters of induction is a strategy to tune the total amount of therapeutic delivered before the population bottleneck occurs and mutants completely replace the original therapeutic bacteria (Fig. 6C). Before mutant takeover, the induction strategy highlighted with a green square in Fig. 6C accomplishes the release of a therapeutic load that is almost 2.5 times higher, when compared to a static induction (Fig. 6D). This result confirms that the dynamic control of gene expression mitigates the effects of mutational escape and can be employed to release higher amounts of therapeutic in lysis-driven bacteria cancer therapy, through multiple boluses.

## Discussion

When designing genetically engineered bacteria for clinical applications, the burden imposed on the metabolism of the bacteria by the synthetic genetic circuit must be taken into account [48,49]. This is of particular relevance when the bacterial therapy is lysis-driven: therapeutic bacteria are under particularly strong negative selection, leading to the occurrence of mutational escape. Various mechanisms of escape, from deletions to deleterious insertions to point mutations, are reported in the literature for kill switches [50].

In this paper we showed, in the case of PFO, that a fundamental limitation of lysis-driven bacteria cancer therapies resides in the emergence of point mutations in the lysis protein and the replacement of the population of therapeutic bacteria with more competitive nonlysing mutants. Previous work to counteract the mutational escape in bacterial therapeutics has focused on optimizing the genetic design to augment stability, i.e., resistance against accumulating genetic mutations [51–53]. For example, it could be investigated whether genomic integration would result in lower mutational escape with respect to our plasmid-borne system.

Using a different approach to mitigate mutational escape, here we proposed an alternative strategy employing a dynamic induction of gene expression. While the recent focus of synthetic biology has been the engineering of various therapeutic payloads to increase efficacy in vivo [54], strategies to demonstrate fine-tuning of the release in vitro have been limited. We developed an ODE model to describe the evolution of mutants and therapeutic bacteria as a function of the intensity and duration of toxin induction. The model is consistent with in vitro data and can serve as a platform to predict the efficacy of the therapy and design optimal strategies of induction. When the toxin is expressed with a single prolonged induction strategy (static), eventually all bacteria expressing a functional form of the protein lyse and the bottleneck removes the selective pressure over emerging or pre-existing nonlysing mutants. Assisted by the model, we suggest that a train of short inductions (dynamic strategy) effectively achieves the release of a higher dose of toxin when compared to the static induction.

We have already mentioned that the SLC accomplishes multiple therapeutic delivery cycles in a microfluidic device, but not in batch culture. In this paper, we suggest that this is possible precisely because the SLC employs a dynamic strategy of expression of the lysis gene, leveraging a quorum-sensing-based genetic circuit and the flow of medium in the microfluidic device preventing excessive accumulation of the autoinducer. Quorum-based induction of lysis in the context of a nutrient-rich and diluting environment (properties of a vascularized tumor [55,56]) is a method to achieve dynamic induction in clinical applications. In this context, see Fig. S12 for a model including a dilution term for AHL, uncovering steady states of the system in longer time frames and the dependence of the fraction of mutants at steady state on the dilution rate. The results of this analysis show that dilution of AHL and bacteria, due to blood flows in the vascularized tumor, when compared to a stagnant medium, would preserve the population of therapeutic bacteria for long time frames and is a powerful asset to achieve dynamic induction in clinical applications. Other methods to achieve dynamic induction could involve the implementation of designed external genetic controllers for the lysis gene. In this context, a major opportunity is the employment of optogenetics modules as controllers [57]. Compared to chemical signaling, using light to regulate and control genetic circuits presents clear advantages, particularly in spatial and temporal modulation [22,58,59]. Near-infrared (NIR)-inducible bacterial optogenetic systems [60,61] are particularly promising for achieving spatially and temporally focused induction in the tumor core, because NIR light effectively penetrates biological tissues [62].

According to our results, after several rounds of dynamic induction, the nonlytic mutant bacteria become dominant and replace the therapeutic strain within a few days. For clinical translation, this issue can be addressed in several ways. First, several attenuated live bacteria strains such as the Food and Drug Administration-approved Vaxcora [63] and Bacillus Calmette-Guérin [64] are unable to colonize patients in the long term and are cleared by the immune system, often within a few days [65]. For *E. coli*, strain SYNB1891 [66,67] contains auxo-trophic mutations limiting growth and enhancing clearance, and has been safely repeatedly dosed intratumorally in patients. Similar attenuation mechanisms could be designed to enhance clearance by the immune system and prevent the negative impact of the remaining nonlytic mutant bacteria that are predicted by our model. We also believe that while mutations arise in several days in vitro, in mouse models and patients, the doubling time of bacteria would be much longer, and mutants may arise at later times [68]. Lastly, while mutations are bound to happen with all live biotherapeutics, regulatory agencies have allowable limits for mutants [69], and we believe that this model can help guide genetic parameter design to achieve this.

Employing a comprehensive set of experimental and computational techniques ranging from microfluidics to sequencing and from mathematical modeling to molecular dynamics, this paper shows that fine-tuning gene expression can address mutation-associated failures of genetic circuits in engineered bacteria for therapeutic applications. Designing bacterial therapeutics with finely regulated release dynamics in space and time is a contribution to the emerging field of cybergenetics [70]. The combination of bacterial therapeutics design and cybergenetics would lead to more insightful considerations over the effects of biological noise on the reliability of the therapies and would provide well-grounded conclusions to scale the therapeutic to in vivo and clinical applications.

When scaling to clinical applications, off-target accumulation of bacteria and toxicity have been previously described as limitations of current bacterial therapies. Strategies that are being actively explored to allow more specific colonization of the tumor while preventing off-target effects are focusing on modulating the immunogenicity of bacteria, exploiting tools from synthetic biology, nanomedicine, and molecular engineering [71]. As an example, previous work characterized a genetic circuit that controls the dynamic expression of a bacterial capsule, endowing *E. coli* with the ability to be overlooked by the immune system in the target diseased tissue, while being cleared from the healthy environments to maintain homeostasis [72]. Given the modularity of genetically engineered bacterial systems, these encapsulation systems could be designed in combination with the proposed controllable circuit for dynamic gene expression within tumors. Another genetic circuit example with dynamic gene expression, the already mentioned SLC, has been demonstrated to be stable in mouse tumors for at least ∼2 weeks [10,18]. While these have yet to be explored clinically, they hold promise for the feasibility of the proposed strategy in the future.

## Data Availability

Data supporting the results of this study are available from the corresponding author upon request.
