# Supplementary material for: Dynamic Gene Expression Mitigates Mutational Escape in Lysis-Driven Bacteria Cancer Therapy
Source: Biodes Res. 2024 Sep 19;6:0049. doi: 10.34133/bdr.0049 (PMC11411163; doi:10.34133/bdr.0049)
Supplement: Supplementary 1 — Figs. S1 to S12 Movies S1 to S3 [file bdr.0049.f1.zip › supplementary_revised.pdf]

SUPPLEMENTARY MATERIALS

Dynamic gene expression mitigates mutational escape in lysis-driven bacteria cancer therapy

Filippo Liguori[1, 2], Nicola Pellicciotta[3,1], Edoardo Milanetti[1,2], Sophia Xi Windemuth[4], Giancarlo Ruocco[1,2], Roberto Di Leonardo[1,3], and Tal Danino[4,5,6*]

[1]Department of Physics, Sapienza University of Rome, Rome, Italy.
[2]Center for Life Nano- & Neuro-Science, Istituto Italiano di Tecnologia, Rome, Italy.
[3]NANOTEC-CNR, Soft and Living Matter Laboratory, Institute of Nanotechnology, Rome, Italy.
[4]Department of Biomedical Engineering, Columbia University, New York, NY, USA.
[5]Herbert Irving Comprehensive Cancer Center, Columbia University, New York, NY, USA.
[6]Data Science Institute, Columbia University, New York, NY, USA.
[*]Address correspondence to: tal.danino@columbia.edu

Fig. S1. Plasmids.
Fig. S2. Dynamics of lysis in the mother-machine.
Fig. S3. Negative control for viability of cancer cells.
Fig. S4. Test of AHL degradation hypothesis.
Fig. S5. Time-courses of the molecular dynamics simulations.
Fig. S6. PFO-induced modulation of the growth rate.
Fig. S7. Fits of experimental growth curves.
Fig. S8. Fitted parameters of the model.
Fig. S9. Characterization of promoter PluxI with GFP reporter.
Fig S10. Model for substrate consumption during plate reader experiments.
Fig. S11. Model for adaptive mutation during plate reader experiments.
Fig. S12. Modeling the system with dilution and for longer time-frames.
Movie S1. Phase contrast video of the lysis in the mother-machine in LB with 100 nM AHL.
Movie S2. Fluorescence video of the lysis in the mother-machine in LB with 100 nM AHL.
Movie S3. Fluorescence video of non-lysing mutants in mother-machine in LB with 100 nM AHL.

1 Plasmids

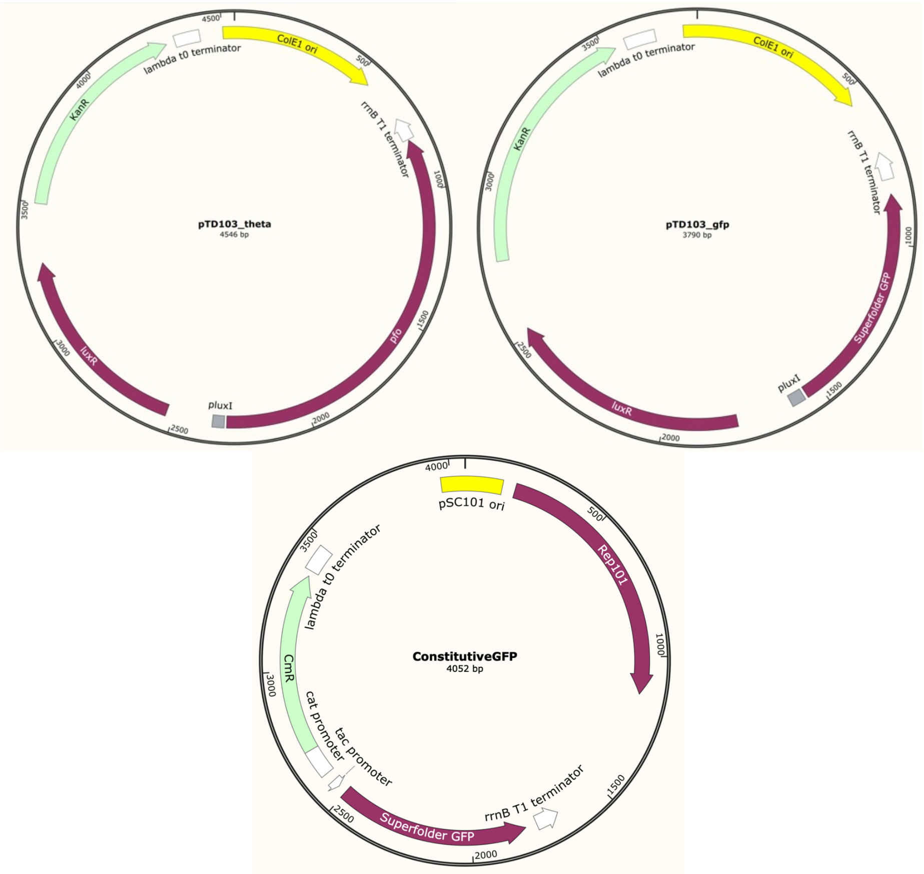

Figure 1: Top left, plasmid encoding the AHL-inducible expression of PFO. Top right, plasmid for AHL-inducible expression of GFP used for the characterization of promoter PluxI. Bottom, plasmid used for the consititutive expression of GFP, used to quantify the release of genetically-encoded cargo upon lysis, and to enable better segmentation of bacteria in the microfluidics device.

31 ## 2 Lysis in mother-machine

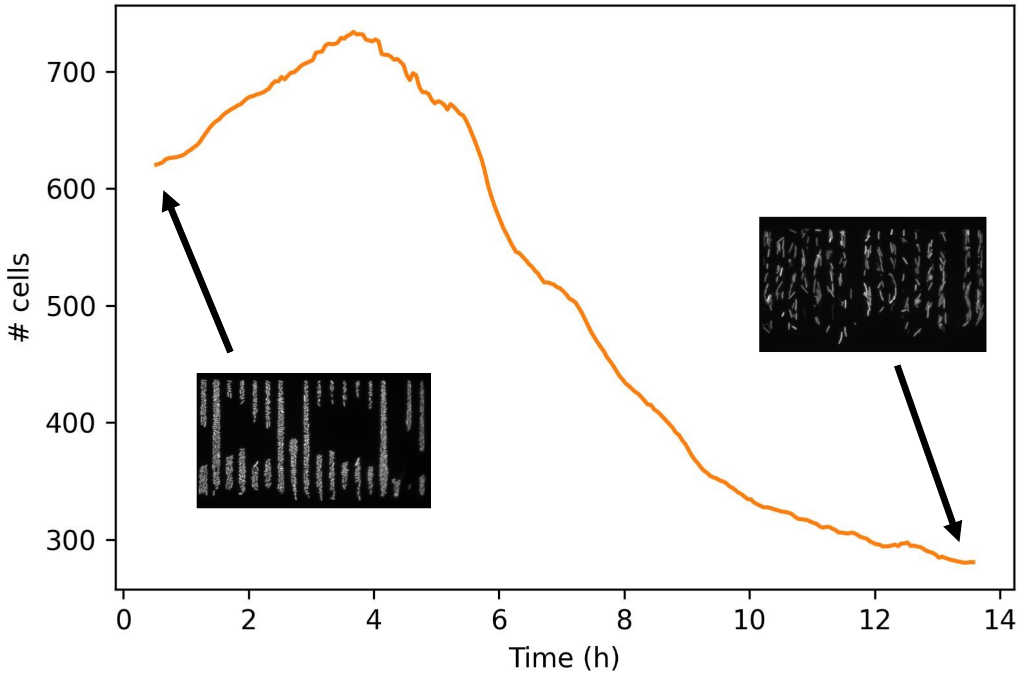

Figure 2: Number of cells as a function of time in a typical mother-machine experiment with 100 nM AHL in the medium. In the insets are shown two example fluorescence snapshots at $t = 0$ and $t = 13h$.

3 Negative control for viablity of cancer cells

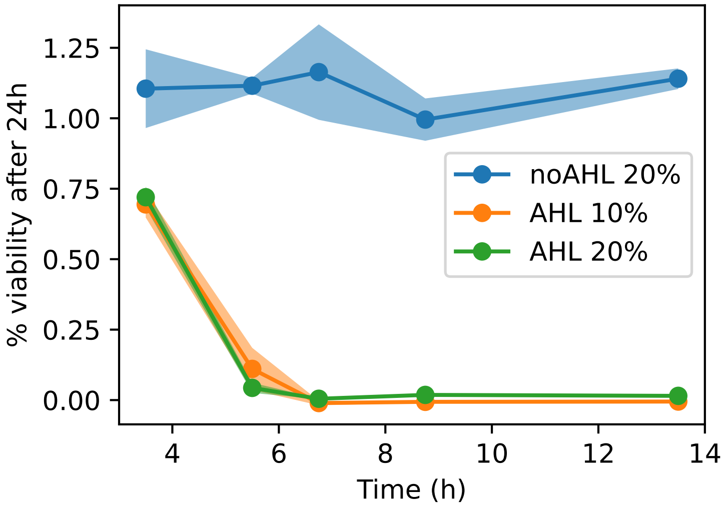

Figure 3: Time evolution of the viability at 24 hours (measured with at MTT assay, see Methods) of CT26 cancer cells cultured with supernatant of therapeutic bacteria for increasing PFO induction times (x axis). The labels stand for: bacteria grown without AHL and cancer cells grown with RPMI supplemented with 20% bacterial supernatant (noAHL 20%), bacteria grown with AHL and cancer cells grown with RPMI supplemented with 10% (AHL 10%) or 20% (AHL 20%) bacterial supernatant. The values are normalized to the viability of cancer cells grown with RPMI supplemented with 20% LB. The shaded area represents the standard deviation of a technical triplicate.

4 Test of AHL degradation hypothesis

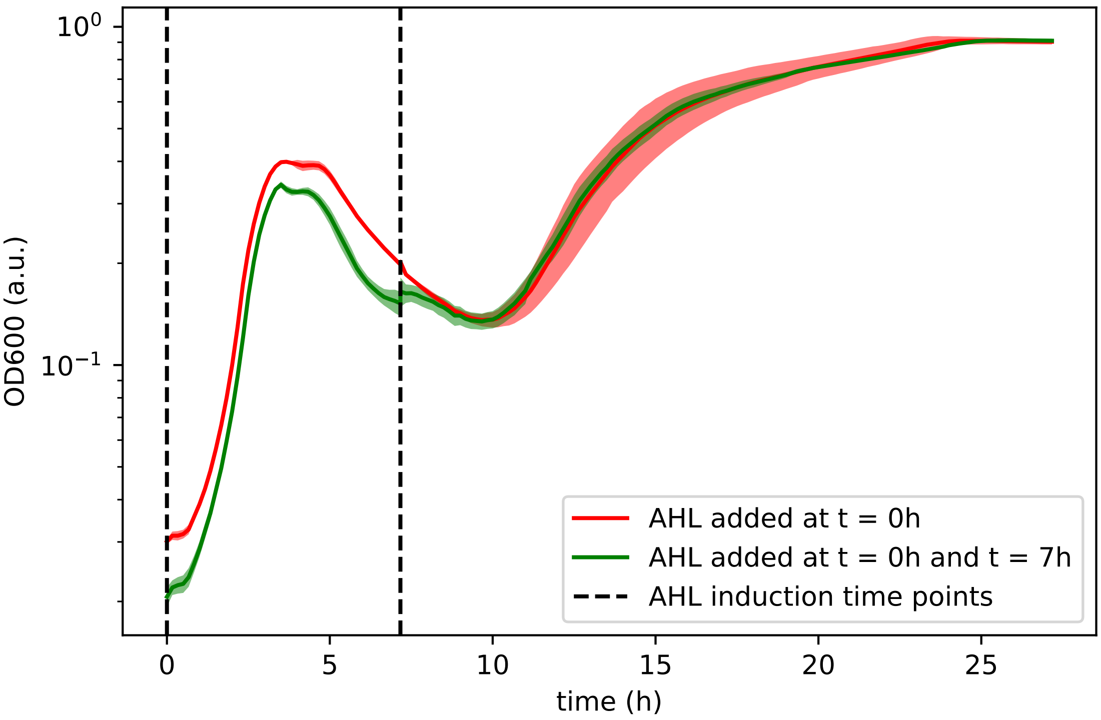

Figure 4: OD600 of MG1655 bearing plasmid pTD103_theta for two different conditions of growth: AHL added only at the beginning of the experiment (red curve) and AHL added twice, at the beginning of the experiment and at a later time point t=7h (green curve). This plot shows that the second growth observed in the OD600 of a typical plate reader experiment is not owing to AHL degradation.

5 Time-courses of the molecular dynamics simulations

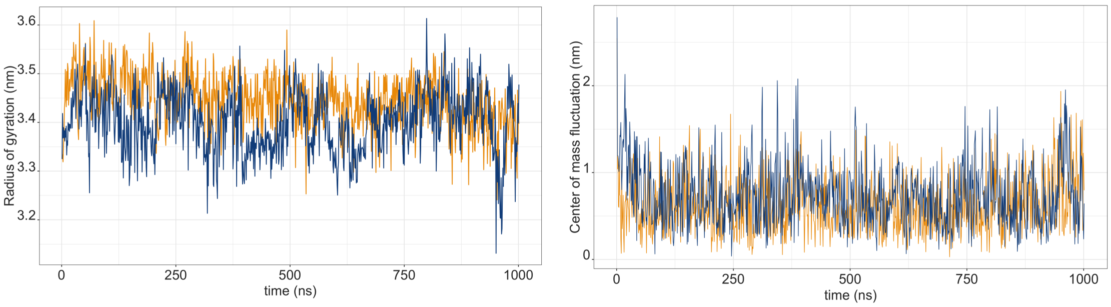

Figure 5: Time evolution of the radius of gyration (left) and center of mass fluctuation (right) in the whole 1 μs time of the molecular dynamics simulations for wild type PFO (blue) and mutant D397N (orange).

6 PFO-induced modulation of the growth rate

The model we employed in the main text (Eq. 1) for the modulation of the growth rate as influenced by the concentration of PFO in the single cell (p) is the following:

$$\gamma_x = \left(\gamma_{max} - \frac{\alpha_0 p^n}{k^n + p^n} \right)^+ \quad (1)$$

where γ_x is the actual growth rate of the bacterial cell, γ_{max} the growth rate the cell would have if it did not express PFO (determined by all other factors like nutrients, crowding etc.) and the growth rate decreases as p increases, being modulated with a Hill function. The formalism $()^+$ represents the positive part of the function in parenthesis, preventing negative growth rates. To validate the model with the experimental data, we compare the experimental growth rates measured in the mother-machine with the model-predicted growth rate for a cell expressing PFO with a constant rate ($\dot{p} = \beta = \text{cost}$) and observe good accordance (Fig. S6b).

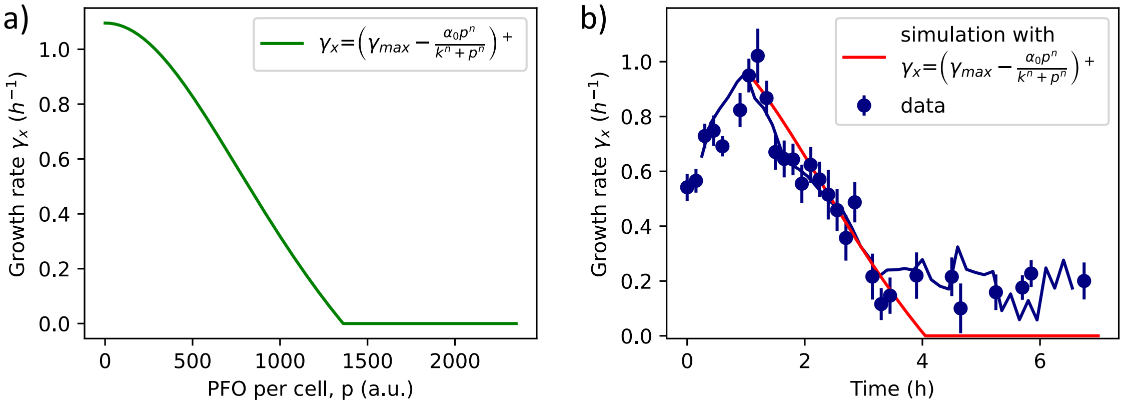

Figure 6: Model of the modulation of the growth rate induced by wild-type PFO. a) Growth rate as a function of PFO concentration in the cell, as modelled in this paper. The values of the parameters are $\gamma_{max} = 1.1\ h^{-1}$ (obtained from Fig. 2d, mean of the values in the orange curve, which is roughly constant), $\alpha_0 = 2.1\ h^{-1}$, $n = 2$, $k = 1300$ a.u. b) Growth rate as a function of time for bacteria expressing PFO with a constant rate $\beta = 3.4$ a.u.h^{-1}. Since the bacteria are growing in a different environment than the plate reader, the parameters can be different, but the form of the modulation is supposed to be the same. Superimposed over the simulation, are the experimental values of the growth rate for therapeutic bacteria expressing PFO in the presence of 100 nM AHL (from Fig. 2d, navy curve).

7 Fits of the experimental growth curves

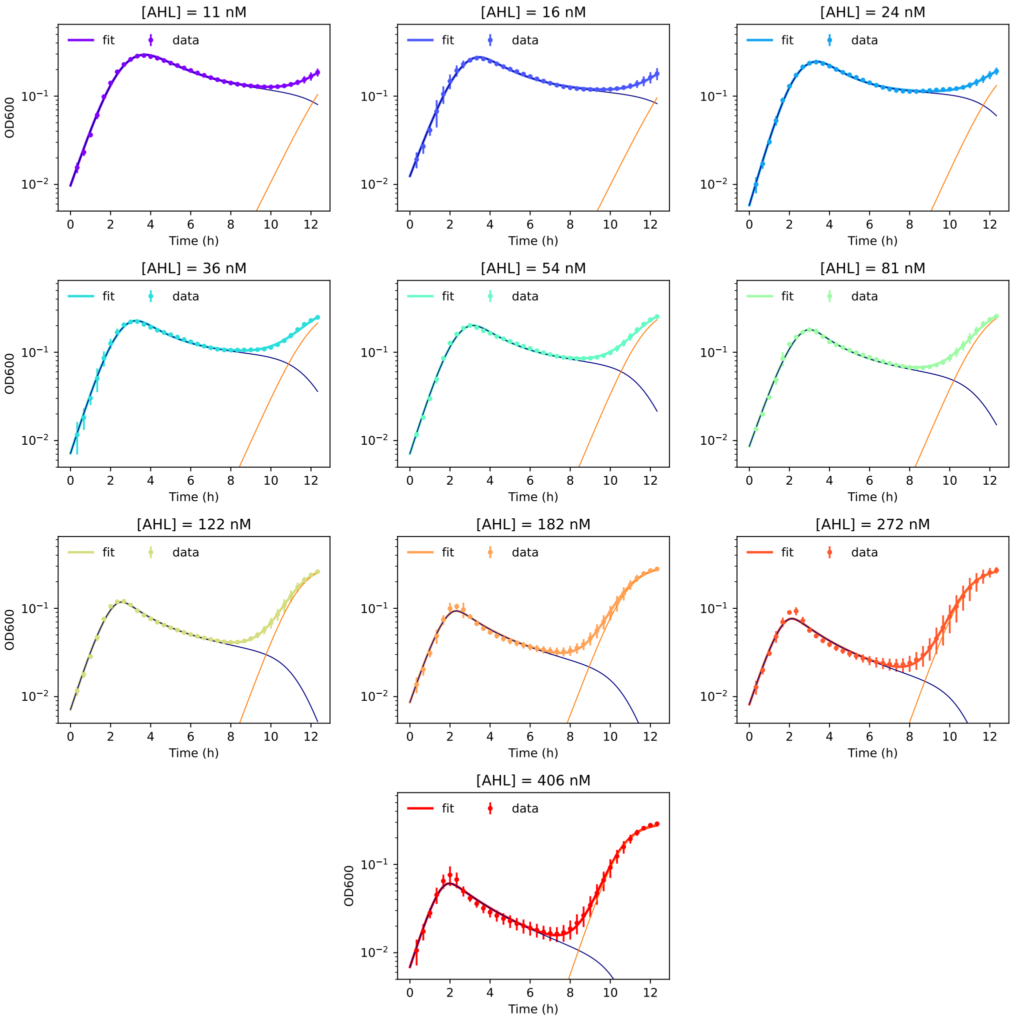

Figure 7: Fits of the growth curves in plate reader with various concentrations of AHL (experimental curves in Fig. 4a, using the same color map). The orange and navy blue thin lines in the plots represent the simulated concentration of mutants (y) and therapeutic bacteria (x) respectively.

8 Fitted parameters of the model

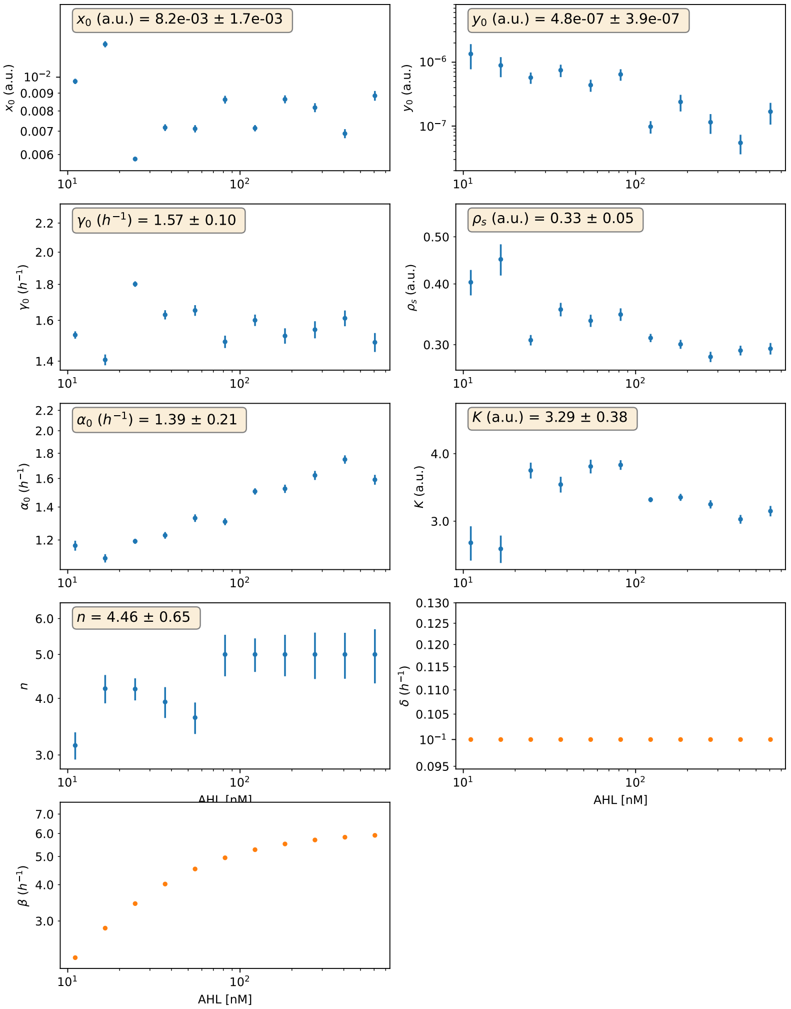

Figure 8: Values of the parameters of the model fitted from the growth curves in plate reader with various concentrations of AHL (fits are shown in Fig. S7). An upper bound of 5 is set for the parameter n. The parameters shown in orange are not fitted but set *a priori* in the simulations. The degradation of intracellular PFO is set *a priori* because the length of the experiment does not reveal such long degradation times. The toxin production rate β is set form the experimental characterization of promoter PluxI shown in Fig. S9.

9 Characterization of promoter PluxI with GFP reporter

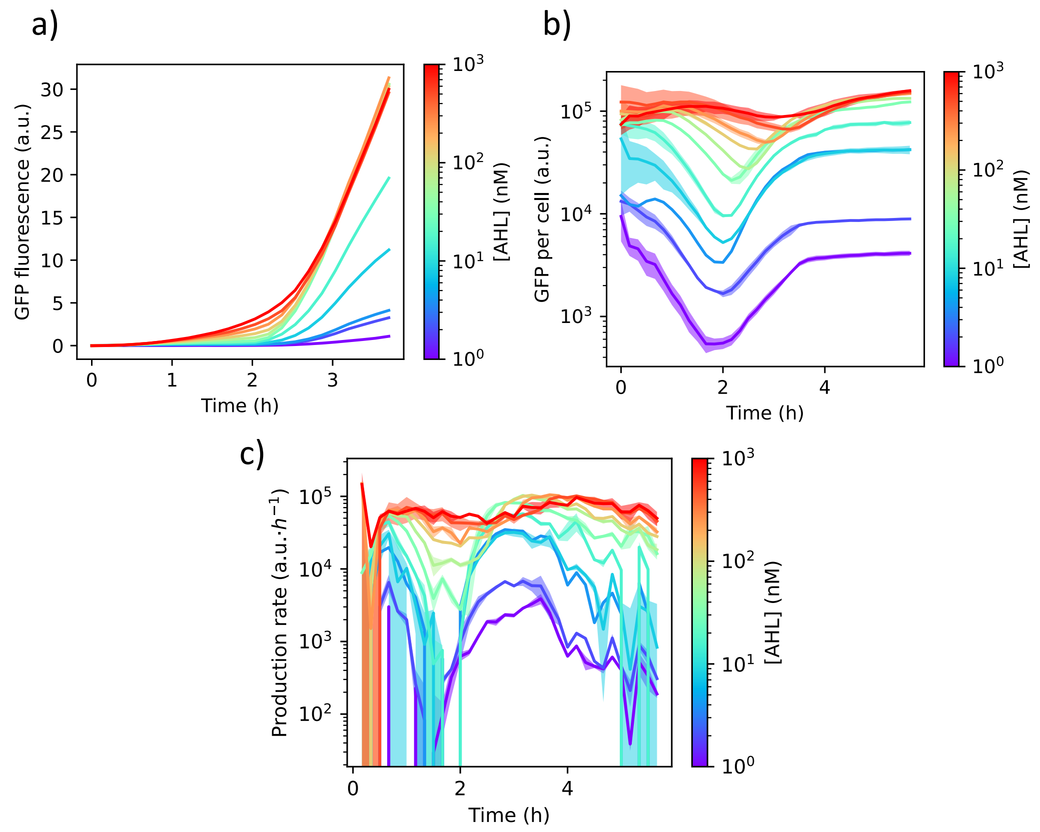

Figure 9: (a) Total GFP as a function of time for MG1655 bearing plasmid pTD103_gfp, with various concentrations of AHL in the medium. (b) GFP per cell computed as [GFP]/OD600, where [GFP] is the total GFP fluorescence of the culture, for the same concentrations of AHL as in a. (c) Protein production rate from promoter PluxI, computed as $\frac{d[\mathrm{GFP}]}{dt}$/OD600. This formula comes from the fact that OD600 is proportional to the total number of bacteria N, and [GFP] can be approximated as the total number of bacteria (N) times the concentration of GFP in the single cell.

10 Model for substrate consumption during plate reader experiments

The model we employed in the main text (Eq. 1) does not account for substrate or nutrient consumption during growth, and the carrying capacity ρ_s in our model is determined exclusively by crowding effects, rather than nutrient consumption. As we discuss in the main text, this is a representative model for tumor-colonizing bacteria in vivo, where blood flows prevent nutrient depletion and the carrying capacity is mainly determined by crowding [1]. In this section, we compare our model with a model where the effects of crowding and substrate consumption are independently accounted for and substrate consumption is explicitly considered in the form of a Monod uptake [2].

The Monod model describes the growth rate at exponential phase as a Hill function of the concentration of nutrients:

$$\gamma_0 = \gamma_0^{(max)} \frac{s}{1+s} \tag{2}$$

where s represents the concentration of the limiting substrate for growth, and the uptake of this substrate is described by the following equation:

$$\dot{s} = -r_s \rho \tag{3}$$

where r_s is the rate of substrate utilization and ρ is the total concentration of bacteria. When we account for this uptake, model in Eq. 1 in the main text can be written as:

$$\begin{aligned}
\dot{x} &= (\gamma - \alpha)x \\
\dot{y} &= \gamma y \\
\dot{s} &= -r_s(x+y) \\
\dot{p} &= \beta([AHL]) - (\gamma - \alpha)^+ p - \delta p \\
\gamma_0 &= \gamma_0^{(max)} \frac{s}{1+s} \\
\gamma &= \gamma_0 \left(1 - \frac{x+y}{\rho_s}\right) \\
\alpha &= \alpha_0 \frac{p^n}{K^n + p^n} \\
\beta &= \frac{\beta_0 [AHL]^m}{k^m + [AHL]^m}
\end{aligned} \tag{4}$$

The selection of parameters r_s and s(t=0) was made in order to maximize agreement with experimental data in Fig. S10a. The plot shows growth curves of the bacteria grown in fresh medium (navy points) and in a consumed medium (orange points). The consumed medium was obtained by growing the bacteria in fresh medium for 18 hours, pelleting the bacteria by centrifugation at 3000 rpm for 5 minutes, collecting the supernatant and passing it through a 0.22 μm filter. From the growth curves, we extracted the growth rate γ_0 by fitting the exponential growth part of the curve (the two values are the red dots in Fig. S10d). Simulating model in Eq. S4 computationally with

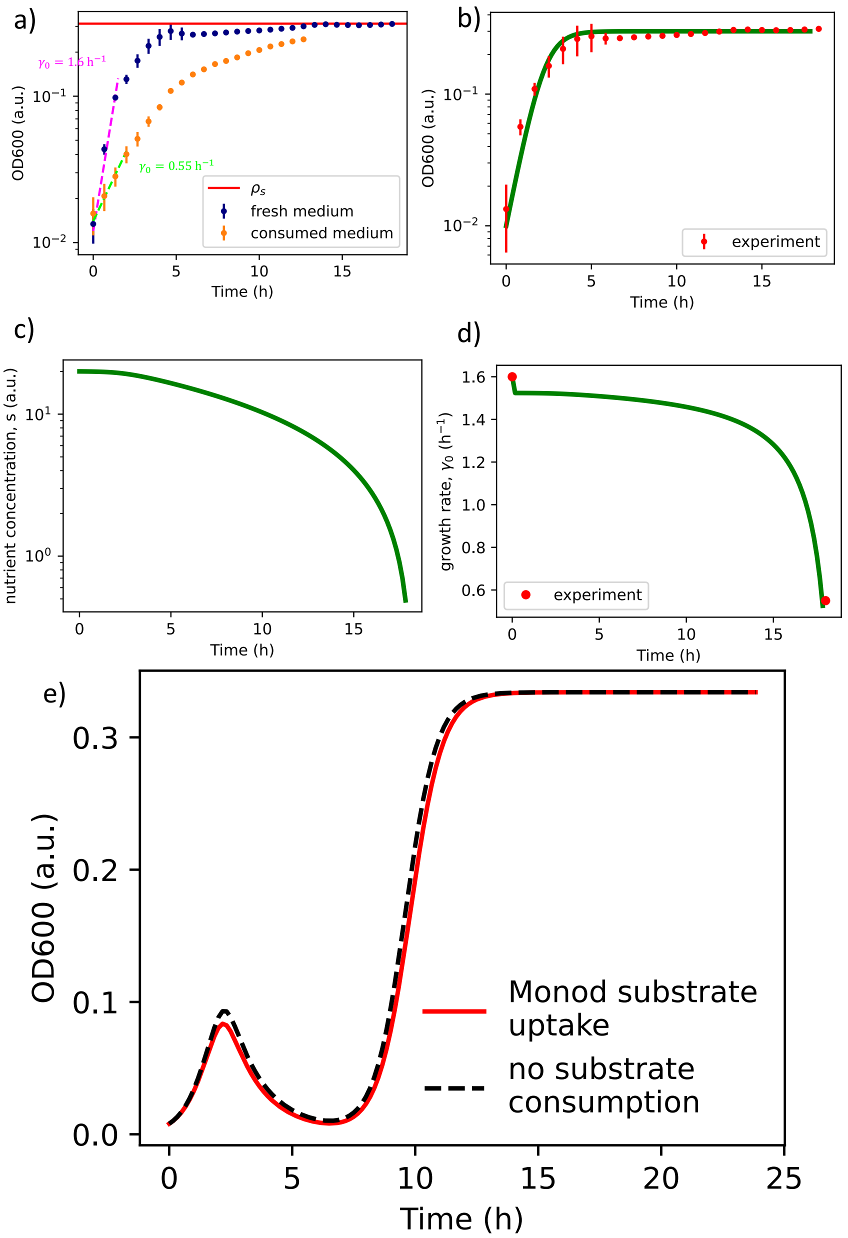

Figure 10: (a) Experimental growth curves of bacteria in fresh and consumed medium, to extract the growth rate at exponential phase for the two cases (magenta and green respectively). (b-d) Simulated time dynamics with model S4 (green line) of OD600 ($\propto$ (x+y)), substrate concentration and growth rate at exponential phase, compared with experimental data (red points, extracted from the growth curves in a). (e) Comparisons between simulations with model in Eq. 1 in the main text (dashed black line, not accounting for substrate consumption) and model in Eq. S4 (red line, accounting for Monod uptake).

parameters $r_s = 4.17$ h^{-1}, s(t=0)= 20 a.u., $\beta([AHL]) = 0$ and the other parameters as specified in the Methods of the main text (Simulations subsection), we obtain values of the OD600 (Fig. S10b) and growth rate at exponential phase γ_0 (Fig. S10d) compatible with experimental data.

By simulating model in Eq. 4 and model in Eq. 1 in the main text with these parameters, for $[AHL]$=100 nM, we observed good similarity between the results of the simulations (Fig. 10e). This discussion shows that accounting for substrate consumption is not critical to reproduce data from plate reader experiments, for the time-frame of the experiments.

11 Model for adaptive mutation during plate reader experiments

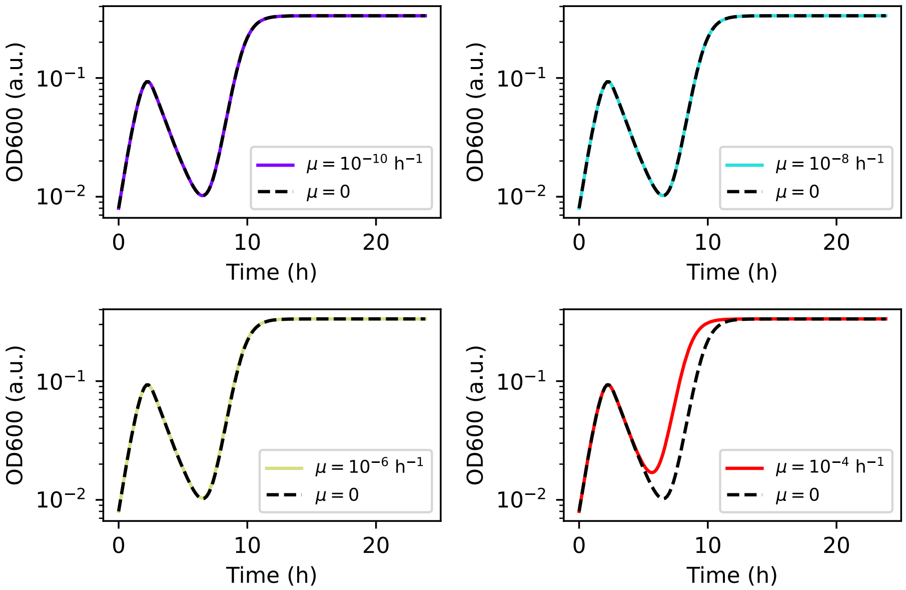

Figure 11: Results of simulations with model in Eq. 1 from the main text (not accounting for adaptive mutation, black dashed line) compared with simulations with model in Eq. S5 (accounting for adaptive mutation, colored lines), for various mutation rates μ. The other parameters are set for all simulations as specified in the Methods of the main text (Simulations subsection), and [AHL] = 100 nM.

As discussed in the main text, we found by fitting the growth curves to our model that the fraction of non-lysing mutants in our frozen glycerol stock (i.e. the initial fraction of mutants in plate reader experiments) is $(7 \pm 6) \cdot 10^{-5}$, probably due to the various overnight cultures between plasmid transformation and stock. Given this relatively high fraction of mutants at time t=0, we considered negligible the dependence of the mutant population y on the therapeutic strain x, governed by the adaptive mutation. In this section we compare our model with a model where the dependence of y from x is explicitly considered, in terms of a mutation rate μ.

$$
\begin{aligned}
\dot{x} &= (\gamma - \alpha - \mu)x \\
\dot{y} &= \gamma y + \mu x \\
\dot{p} &= \beta([AHL]) - (\gamma - \alpha)^{+}p - \delta p \\
\gamma &= \gamma_0\left(1 - \frac{x+y}{\rho_s}\right) \\
\alpha &= \alpha_0\frac{p^n}{K^n + p^n} \\
\beta &= \frac{\beta_0[AHL]^m}{k^m + [AHL]^m}
\end{aligned} \tag{5}
$$

We simulated this model for various values of the mutation rate, ranging from 10^{-10} to 10^{-4} h^{-1} (Fig. S11), and no significant difference from the model not accounting for adaptive mutation (μ=0) is observed for mutation rates $\mu \leq 10^{-6}$ h^{-1}.

The adaptive mutation rate reported in the literature for *E. coli* is 10^{-11}-10^{-8} errors per base-pair per round of replication [3]. Since the length of the *pfo* gene is $\approx$ 1 kb and the replication time is $\approx$ 1 h, a plausible value of μ is of the order 10^{-7} h^{-1}. Therefore, this analysis supports the claim that the dependence of the mutant population y on the therapeutic strain x, governed by the adaptive mutation, can be neglected for the time-frame of the plate reader experiments.

12 Modeling the system with dilution and for longer time-frames

In this section, we discuss an expansion of the model in Eq. 1 in the main text, adding a dilution term (D) due to the presence of flows or other factors depleting AHL and bacteria. This refined model is the following:

$$\begin{aligned}
\dot{x} &= (\gamma - \alpha - \mu - D)x \\
\dot{y} &= (\gamma - D)y + \mu x \\
\dot{p} &= \beta([AHL]) - (\gamma - \alpha)^{+}p - \delta p \\
[A\dot{H}L] &= -D \cdot [AHL] \\
\gamma &= \gamma_0 \left(1 - \frac{x+y}{\rho_s}\right) \\
\alpha &= \alpha_0 \frac{p^n}{K^n + p^n} \\
\beta &= \frac{\beta_0 [AHL]^m}{k^m + [AHL]^m}
\end{aligned} \tag{6}$$

The dynamics of the autoinducer is determined solely by the dilution rate and the concentration added externally at $t = 0$, since bacteria cannot synthesize AHL. Simulating this model for the usual values of all parameters, as fitted from the growth curves in Fig. S7, $\mu = 10^{-7}$ and [AHL](t=0)=100 nM, we varied the parameter D to uncover steady states of the system for longer time-frames than those explored in our plate reader experiments (time-frame t_{max} of more than 4 days, Fig. S12a), in a condition reminiscent of the *in vivo* environment. As discussed in the main text, in fact, adding a dilution term is a representative model for tumor-colonizing bacteria *in vivo*, where blood flows in the tumor prevent nutrient depletion and dilute metabolites or other products of the bacteria. These steady states exhibit a decreasing fraction of mutants f for increasing dilution rates (Fig. S12b).

The results of this analysis show that dilution of AHL and bacteria, due to blood flows in the vascularized tumor [4, 5], when compared to a stagnant medium, would preserve the population of therapeutic bacteria for long time-frames and is a powerful asset to achieve *dynamic* induction in clinical applications.

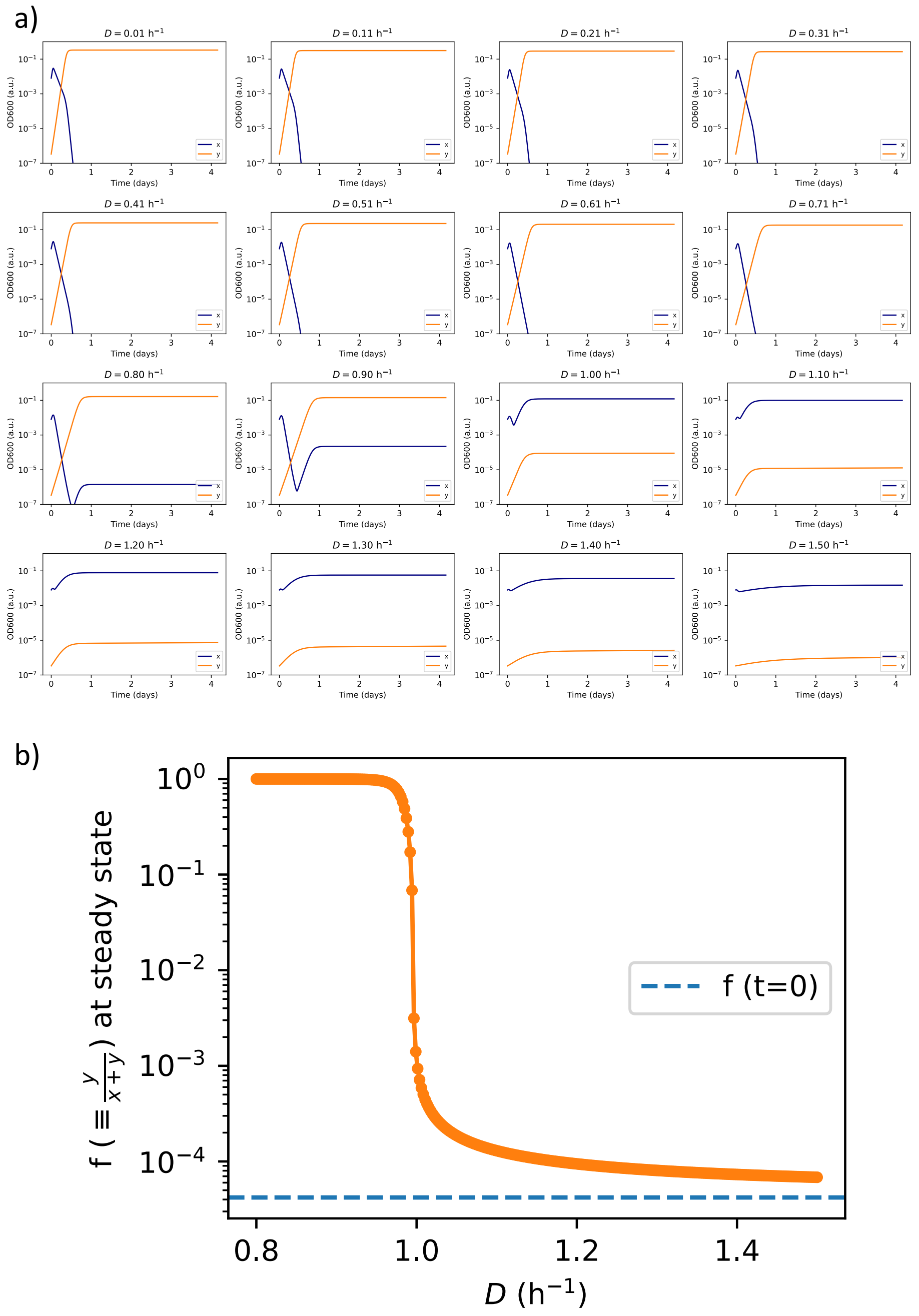

Figure 12: Results of simulations with model in Eq. S6 (accounting for adaptive mutation and dilution of AHL and bacteria), for various dilution rates D. (a) Time-dynamics for a time-frame of approximately 4 days of the concentration of therapeutic bacteria (x) and mutants (y), uncovering steady states exhibiting a decreasing fraction of mutants f for increasing dilution rates. (b) Fraction of mutants at the steady state as a function of the dilution rate after a time-frame t_{max} of 4 days.

13 Description of supplementary videos

Movie S1 shows the phase contrast frames acquired in a typical mother-machine experiment, where 100 nM AHL is present in the medium. Once all bacteria in the channels have lysed, the flow is stopped and the channels are filled again by remaining bacteria, revealing the emergence of non-lysing mutants. Movie S2 and S3 show the fluorescence frames of the first and second part of the same experiment. The videos are played at 12 frames/s. The time is indicated in the top-left corner. The mean growth rate of all cells as a function of time is shown in Fig. 2d for therapeutic bacteria (Movie S2) and mutants (Movie S3).

References

1. Vaupel P, Kallinowski F, and Okunieff P. Blood flow, oxygen and nutrient supply, and metabolic microenvironment of human tumors: a review. Cancer research 1989;49:6449–65.
2. Koch AL. The Monod model and its alternatives. In: *Mathematical modeling in microbial ecology.* Springer, 1998:62–93.
3. Fijalkowska IJ, Schaaper RM, and Jonczyk P. DNA replication fidelity in Escherichia coli: a multi-DNA polymerase affair. FEMS microbiology reviews 2012;36:1105–21.
4. Sznol M, Lin SL, Bermudes D, Zheng Lm, King I, et al. Use of preferentially replicating bacteria for the treatment of cancer. The Journal of clinical investigation 2000;105:1027–30.
5. Chaplain MA, McDougall SR, and Anderson A. Mathematical modeling of tumor-induced angiogenesis. Annu. Rev. Biomed. Eng. 2006;8:233–57.
